# Inhibitory effects of compounds from the roots of *Potentilla longifolia* on lipid accumulation

**DOI:** 10.1371/journal.pone.0238917

**Published:** 2020-09-09

**Authors:** Shengxi Lin, Xiaoyan Zhao, Yunpeng Sun, Huan Liu, Mingyang Shang, Jinyan Gong, Qianqian Ma, Guangchun Piao, Haidan Yuan

**Affiliations:** 1 College of Pharmacy, Yanbian University, Yanji, Jilin, China; 2 Key Laboratory of Natural Resources of Changbai Mountain & Functional Molecules, Ministry of Education, Yanbian University, Yanji, Jilin, China; Luleå University of Technology, SWEDEN

## Abstract

*Potentilla longifolia* is a kind of Chaoyao medicine, which is a branch of traditional Chinese medicine. The plant is often referred to as *ganyancao* or *ganyearmcao*, which means that it has a significant therapeutic effect on liver inflammation. In previous experiments, we found that a water extract of *ganyearmcao* inhibited lipid accumulation. In the present study, we isolated one new (ganyearmcaoone A, **1**) and eight known compounds (**2**–**9**) from a water extract of the dried roots of *ganyearmcao*; all of the compounds were isolated for the first time from this medicinal plant. We elucidated the chemical structures of these compounds using comprehensive analyses of HR-ESI-MS and 1D, 2D NMR. We evaluated the inhibitory effects of the nine compounds on lipid accumulation in 3T3-L1 cells; we did so using photographic and quantitative assessments of the lipid content with oil red O staining and by measuring triglyceride levels. Compared with the control, compounds **6** and **9** significantly inhibited differentiation of 3T3-L1 cells and lipid accumulation. Compound **1** showed potential inhibitory effects on lipid accumulation. Molecular docking results indicated that compounds **6** and **9** may efficiently bind to AMPK and its downstream kinase (SCD1), thereby inhibiting lipid accumulation. Our results demonstrate that *ganyearmcao* and its components may play an important role in treating diseases related to lipid accumulation in the future.

## Introduction

For thousands of years, human health has been closely related to natural products and traditional medicines in many parts of the world. Likewise, efforts to treat and prevent diseases using natural products and traditional medicines are evident globally. Since morphine was first isolated from opium in 1805, there has been ongoing exploration of natural products and traditional medicines. Modern medicine has benefited considerably from that experience [1, 2]. Traditional Chinese medicine (TCM) includes the traditional medicines of all nationalities in China [3].Chaoyao medicine, which is a branch of TCM, embodies the valuable experience accumulated by the Chaoxianzu (an ethnic minority in China) in the long-term application of the traditional medicine. *Potentilla longifolia* Wild. Ex Schlecht is a kind of Chaoyao medicine. Both its roots and aerial parts can be used in medicine. In the area where it is used in China, the herb is often referred to as *ganyearmcao* or *ganyancao*, which means that it has significant therapeutic effects on liver inflammation [4, 5].

Excessive fat accumulation has lead to fatty liver, obesity, and many other diseases such as hypertension, hyperlipidemia, and diabetes [6–8]. Nonalcoholic fatty liver disease (NAFLD) has gradually become one of the leading causes of chronic liver disease in many developed countries. The high prevalence of NAFLD in the general population underlines its clinical importance [7, 9]. NAFLD is a kind of disease caused by excessive accumulation of fat in liver tissue. Even when a patient usually takes little or no alcohol, the fat accumulation is more than 5% of the weight of the liver, or more than one third of hepatocytes contain too much fat histologically. Pathologically, it may develop into nonalcoholic simple fatty liver, nonalcoholic fatty hepatitis, and related fatty liver fibrosis and cirrhosis. NAFLD may be caused by multiple factors including insulin resistance, lipid metabolism disorder, and oxidative stress, etc [10, 11]. Obesity results from excessive or abnormal fat accumulation, which impairs health and increases mortality. Obesity is often regarded as a chronic disease that requires constant efforts to return to a normal weight [12, 13]. By 2030, the prevalence of diabetes in the world will rise to 11.4% of the total population: that development may be attributed to the rapid economic development, improved living standards, and the aging population [14, 15]. In recent years, researchers have been eager to seek inspiration in this regard from medicinal plants (especially those having considerable application experience) and derive bioactive natural products from them to treat fatty liver.

AMP-activated protein kinase (AMPK) is closely involved in the metabolism of fats and carbohydrates. After activation, AMPK can inhibit the expressions of sterol regulatory element binding protein (SREBP1c), peroxisome proliferator-activated receptor γ (PPARγ), CCAAT/enhancer-binding protein (C/EBPα), and their downstream proteins. Examples of these proteins are stearoyl-CoA desaturase-1 (SCD1) and fatty acid synthase (FAS); the latter is involved in the biosynthesis of triglycerides (TGs) and fatty acids as well as in adipocyte maturation, thereby suppressing adipogenesis [16, 17]. Furthermore, molecular docking research has often been used to examine the position of a compound at any protein-binding site [18, 19]. Molecular docking research is a computer simulation methodology that can be applied in optimization problems and automatically determining the conformation of a protein-ligand complex [20].

In our previous experiments, a water extract of *ganyancao* had the effect of inhibiting lipid accumulation. Because both its roots and aerial parts have been used in medicine, it is necessary to seperate the active ingredients from the roots of *ganyancao*. In the present study, we separated the components from the plant’s roots at first; we measured their activities in inhibiting lipid accumulation and studied the related mechanisms.

## Materials and methods

### General experimental procedures

The roots of *Potentilla longifolia* were extracted with H_2_O three times, then the resulting extract was subjected to various seperation methods such as D101 macroporous resin, silica gel and reverse column chromatography, HPLC, etc., to obtain nine compounds. The structures of the nine compounds were identified by using comprehensive analyses of HR-ESI-MS and 1D, 2D NMR. Then the inhibitory effects of the nine compounds on lipid accumulation were evaluated in 3T3-L1 cells by using photographic and quantitative assessments of the lipid content with oil red O staining and measuring triglyceride levels. At last, molecular docking of compounds **6** and **9** (two most active compounds) were carried out to evaluate their potential mechanisms concerning their binding abilities with AMPK and its downstream kinase SCD1.

### Techniques and materials for separation and identification

The NMR spectra were recorded using Bruker AV500/300MHz spectrometer (Bruker, Fallanden, Switzerland). High-resolution electrospray ionisation mass spectra (HR-ESI-MS) were obtained using a Bruker microTOF QII mass spectrometer (Bruker Daltonics, Fremont, CA, USA). Sephadex LH-20 was purchased from GE Healthcare (USA). Optical rotation was measured using a Rudolph Autopol I automatic polarimeter (Rudolph Research Analytical, Hackettstown, NJ, USA). Silica gel (200–300 mesh) was purchased from Qingdao Haiyang Chemical Co., Ltd, Qingdao, P. R. China. TLC was performed using precoated silica gel 60 RP-18 F254s glass and aluminum plates (200×200 mm, Merck, Germany). ODS (50 μm) was obtained from YMC(Japan). All other chemicals and solvents were analytical grade.

### Cells and reagents

The 3T3-L1 cells were purchased from ATCC (Manassas, VA, USA). Dulbecco’s modified Eagle’s medium (DMEM), fetal calf serum (FCS), fetal bovine serum (FBS), and penicillin−streptomycin were purchased from Gibco by Life Technologies (Grand Island, NY, USA). The TG assay kit was purchased from Nanjing Jiancheng Bioengineering Institute, China.

### Plant material

The roots of *Potentilla longifolia* Wild. Ex Schlecht. was acquired in Changbai Mountain, Jilin Province, China, in October 2016. The sample was authenticated by Prof. HZ Lv of College of Pharmacy, Yanbian University (voucher specimen: ID-2016053, stored in Chaoyao Herbarium of Yanbian University). After being dug out, the roots were washed with water and air-dried and then stored in dry space. One month later, the roots were cut to about 2cm with a chopper and then extracted.

### Extraction and isolation

The roots of *Potentilla longifolia* (10 kg) were extracted with H_2_O (2 h×3). The resulting extract (1578 g) was subjected to D101 macroporous resin column chromatography with H_2_O, 25%, 50%, 75% and 95% EtOH successively to give H_2_O (434g) (A), 25% (526 g) (B), 50% (139 g) (C), 75% (5.2 g) (D), 95% EtOH (2.8g) (E) fractions.

The 95% EtOH (2.8 g) fraction was subjected to silica gel column chromatography with a gradient of petroleum ether-EtOAc (20:1, 10:1, 5:1, 3:1), and CH_2_Cl_2_-MeOH (20:1, 10:1, 5:1, 100% MeOH) successively to give 23 subfractions (Fr. E-1^__^23). Fr. E-8 (44 mg) was subjected to HPLC with a gradient of MeOH-H_2_O (1:1–2:1–0:1) to obtain compound **2** (4.4mg) and **3** (3.4 mg). Fr. E-13 (94.5 mg) was subjected to reverse column (ODS-A) chromatography with a gradient of MeOH-H_2_O (1:2–1:1–3:2–2:1–1:0) to yield compound **1** (6.0 mg). Fr. E-14 (84.8 mg) was subjected to reverse column (ODS-A) chromatography with a gradient of MeOH-H_2_O (1:1, 3:2, 2:1, 5:2, 1:0) to obtain 18 subfractions (E-14-1^__^18). E-14-15 (13.7 mg) was separated using silica gel column chromatography with a gradient of petroleum ether-EtOAc (5:1, 3:1) to yield compound **7** (1.5 mg). Fr. E-14-12 (7.3 mg) was subjected to HPLC with a gradient of MeOH-H_2_O (2:3, 1:1, 2:1, 1:0) to yield compound **6** (1.6 mg). Fr. E-11 (72.8 mg) was subjected to reverse column (ODS-A) chromatography with a gradient of MeOH-H_2_O (2:3, 1:1, 3:2, 2:1, 5:2, 3:1, 4:1, 1:0) to obtain compound **8** (1.8 mg).

The 75% (5.2 g) fraction was subjected to silica gel column chromatography with a gradient of CH_2_Cl_2_-MeOH (20:1, 10:1, 5:1, 3:1), and CH_2_Cl_2_-MeOH-H_2_O (5:1:0.1, 3:1:0.1, 1:1:0.1) to give 15 subfractions (Fr. D-1^__^15). Fr. D-10 (72.8 mg) was subjected to silica gel column and reverse column (ODS-A) chromatography successively to obtain compound **4** (10.1 mg). Fr. D-4 (84 mg) was subjected to silica gel column with a gradient of CH_2_Cl_2_-MeOH (35:1, 0:1), to obtain compound **9** (10.4 mg).

The 50% (139 g) fraction was dispersed in H_2_O (5 l) and extracted successively with EtOAc, and n-BuOH respectively, yielding EtOAc (11.3 g), n-BuOH (12.9 g), and water (92.5 g) fractions. The EtOAc fraction (11.3 g) was subjected to silica gel column chromatography with a gradient of CH_2_Cl_2_-MeOH (20:1, 10:1, 5:1, 2:1, 1:1, 0:1) to give 10 subfractions (Fr. C-1^__^10). Fr. C-4 (910 mg) was subjected to silica gel column, reverse column (ODS-A) and Sephadex LH-20 column chromatography successively to obtain compound **5** (13.2 mg).

### Cell culture and cytotoxicity assay

The 3T3-L1 cells were cultured in DMEM containing 10% FCS, 100 μg/ml streptomycin and 100 unit/ml penicillin at 37°C in an atmosphere of 5% CO_2_. 3 × 10^4^ 3T3-L1 cells per well were cultured in 96-well plates and treated with the nine compounds at the concentrations of 0, 10, 20, 40, or 80 μM for 96 h respectively for the cytotoxicity assay. Three parallel wells were set for each concentration. The cytotoxicities of the nine compounds were determined by the MTT assay. The absorbance was measured at 540 nm to determine the numbers of living cells in wells.

The 3T3-L1 cells (5 × 10^5^ cells per well) were cultured in 6-well culture plates. The 3T3-L1 cells were divided into a normal control (CON) group, a differentiated control treated with differentiation medium (DM) group, a differentiated positive control treated with DM plus pioglitazone (PIO) group, nine compound treatment groups treated with the each of nine compounds, respectively. The nine compound treatment groups were treated with 40 μM of compounds **1**, **3**, **5**, **7**, **8** and **9**, 20 μM of compounds **2**, **4** and **6**, respectively.

When the cells were incubated until confluence (day 0), they were exposed to DM I (DMEM, 5% FBS, 10 μg/ml insulin, 1 mM dexamethasone, and 0.5 mM 3-isobutyl-1-methylxanthine) for 4 days (day 4); then, except for the CON group, the cells were exposed to DM II (DMEM containing 5% FBS and 10 μg/ml insulin) for 2 more days (day 6); and then, except for the CON group, the cells were exposed to DM III (DMEM containing 5% FBS) for 2 more days (day 8). From day 0 to day 4 during adipocyte differentiation, the cells in nine compound treatment groups were treated with corresponding compounds with corresponding concentrations.

### Oil-Red O staining

After removal of the medium in the 6-well plate, the differentiated cells were washed twice with phophate-buffered saline (PBS) and fixed with 10% formalin for 1 h. The cells were then stained with 1 ml Oil-Red O solution for 2 h to observe the differentiation of 3T3-L1 cells. The pictures were taken under an Olympus microscope. After that, 6-well plates were treated with isopropanol, and the absorbances were measured at 540 nm to evaluate the accumulation of lipids.

### Measurement of the TG level

The 3T3-L1 cells were lysed in lysis buffer which included 25 mM sucrose, 20 mM Tris−HCl, 1 mM EGTA, and 1 mM EDTA, and then the cells were collected and centrifugated at 13000 rpm for 15 min. A TG assay kit was used to measure the levels of TG in accordance with the instructions of the manufacturer. A Bio-Rad protein assay reagent (Bio-Rad Laboratories) was used to determine the concentration of protein in accordance with the manufacturer’s instructions.

### Molecular docking studies

The crystal structures of the kinases of SCD1 (PDB ID:4YMK) and AMPK (PDB ID:5T5T) were downloaded from RCSB Protein Data Bank and the 3D structures of compounds were transferred by Chem3D 14.0.0.117. Then, the proteins and compounds were prepared by Discovery Studio 4.0. The molecular docking study were performed using the Libdock in Discovery Studio 4.0.

### Statistical analysis

All data were expressed as a mean ± standard error, and differences between groups were analyzed by one way ANOVA analysis followed by Student−Newman−Keuls. Each value was the mean of three separate experiments, and mean values were considered significantly different when p< 0.05. Calculations were performed using the SigmaStat software version 3.5 (Systat Software, Inc., Chicago, IL, USA).

## Results

### Chemical structures of the nine compounds

**Fig 1:**
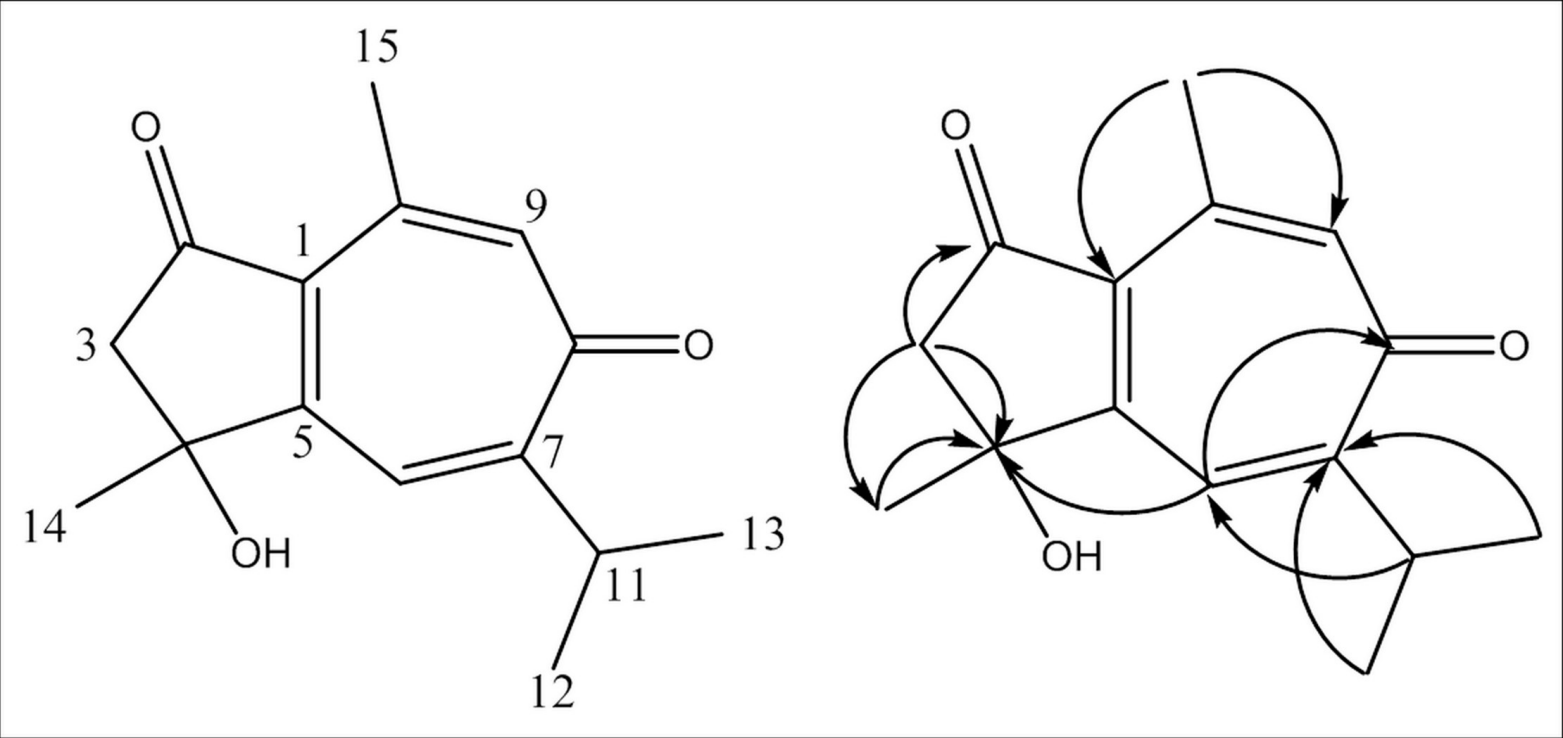
The chemical structure and key HMBC (H→C) correlations of compound 1. HMBC = Heteronuclear Multiple Bond Correlation.

**Fig 2:**
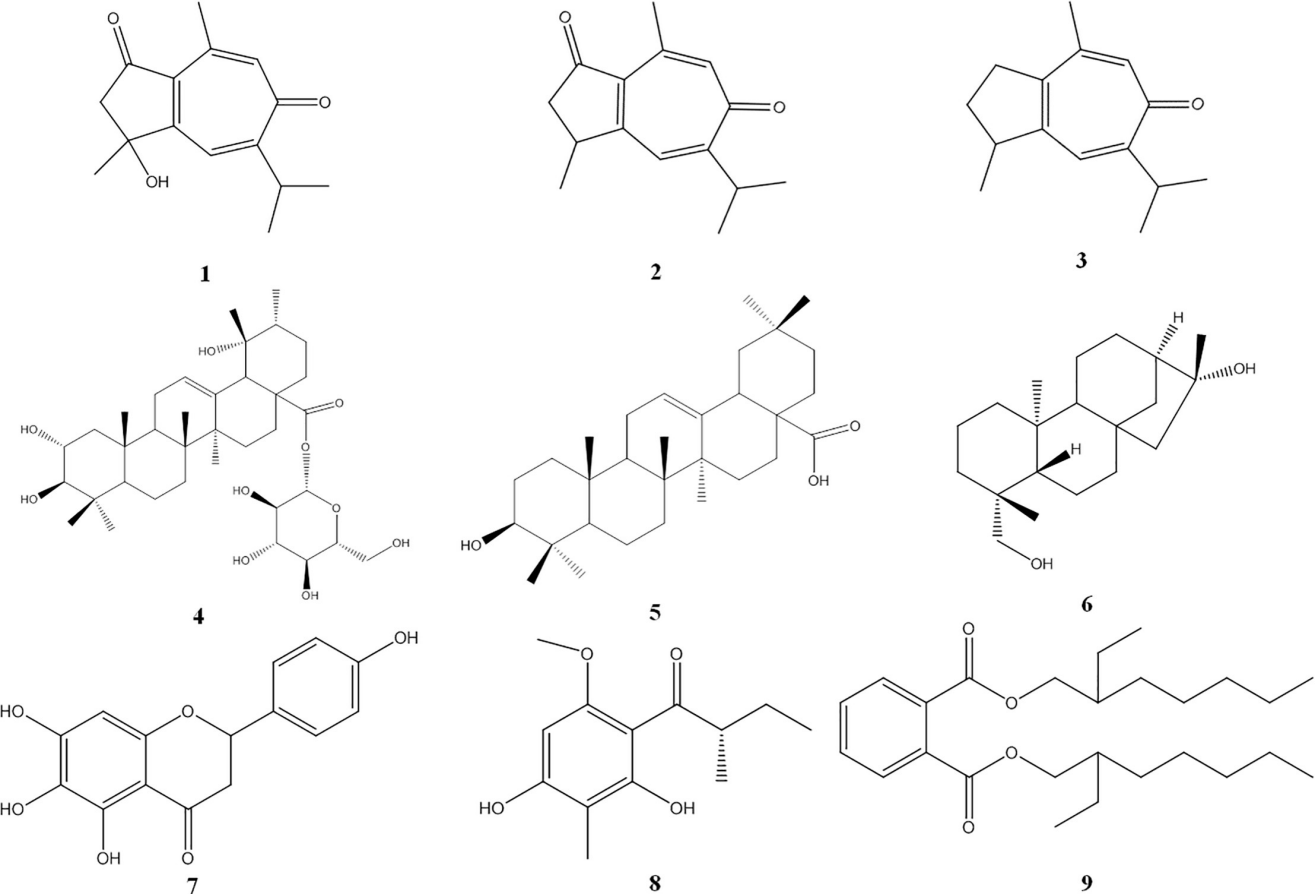
The chemical structures of compounds 1–9 isolated from *Potentilla longifolia*.

We isolated compound **1** as a white amorphous powder. We established its molecular formula as C_15_H_18_O_3_ by [M+H]^+^ peak at *m*/*z* 247.1325 (calculated for C_15_H_19_O_3_, 247.1329) in the HR-ESI-MS spectrum (S5 Fig in S1 File). The ^1^H NMR spectrum contained the following: two olefinic protons at δ_H_ 7.66 (1H, s, H-6) and 6.86 (1H, d, *J* = 1.1 Hz, H-9); one methine proton at δ_H_ 3.41 (1H, dt, *J* = 14.3, 6.9 Hz, H-11); a methylene group at δ_H_ 2.76 (2H, s, H-3); and four methyls at δ_H_ 2.54 (3H, d, *J* = 1.1 Hz, H-15), 1.56 (3H, s, H-14), 1.27 (3H, d, *J* = 6.9 Hz, H-12), and 1.24 (3H, d, *J* = 6.9 Hz, H-13) (Table 1, S1 Fig in S1 File). The ^13^C NMR spectrum revealed 15 carbon resonances, which included the following: two carbonyl carbons at δ_C_ 204.3 (C-2) and 188.0 (C-8); six olefinic carbons at δ_C_ 169.1 (C-5), 165.8 (C-7), 144.9 (C-10), 140.2 (C-9), 136.2 (C-1), and 128.4 (C-6); an oxygenated quaternary carbon at δ_C_ 75.2 (C-4); a methylene carbon at δ_C_ 52.6 (C-3); one methine carbon at δ_C_ 32.3 (C-11); and four methyls at δ_C_ 28.7 (C-14), 23.6 (C-15), 22.5 (C-12), and 22.4 (C-13) (Table 1, S2 Fig in S1 File). Parts of the ^1^H and ^13^C NMR spectra of compound **1** were similar to that of pernambucone—a compound with an unusual seven-membered ring structure [20]. That observation was confirmed by the 2D NMR data of compound **1**. The key HMBC correlations among H-3 (δ_H_ 2.76), H-6 (δ_H_ 7.66), H-14 (δ_H_ 1.56), and C-4 (δ_C_ 75.2) (Fig 1, S4 Fig in S1 File) suggested that a hydroxy group should be located at C-4. Consequently, we identified the structure of compound **1** as 4-hydroxy-1(5),6(7),9(10)-guaiatrien-2,8-dione (ganyearmcaoone A) (Fig 2).

**Table 1:** ^1^H NMR and ^13^C NMR data of compound 1 (CD_3_OD, *J* in Hz).

Position	δ_H_	δ_C_
1	—	136.2
2	—	204.3
3	2.76, s	52.6
4	—	75.2
5	—	169.1
6	7.66, s	128.4
7	—	165.8
8	—	188.0
9	6.86, d (1.1)	140.2
10	—	144.9
11	3.41, dt (14.3, 6.9)	32.3
12	1.27, d (6.9)	22.5
13	1.24, d (6.9)	22.4
14	1.56, s	28.7
15	2.54, d (1.1)	23.6

The known compounds **2**–**9** have been identified as follows: pernambucone (**2**) [21]; orobanone (**3**) [21]; rosamultin (**4**) [22]; oleanolic acid (**5**) [23]; ent-16*β*,19-dihydroxykaurane (**6**) [24]; carthamidin (**7**) [25]; 1,5-dihydroxy-2-(2’-methylbutanoyl)-3-methoxy-6-methylbenzene (**8**) [26]; and bis (2-ethylheptyl) phythalate (**9**) [27]. Their corresponding chemical structures appear in Fig 2.

### Effects on viability of 3T3-L1 cells

**Fig 3:**
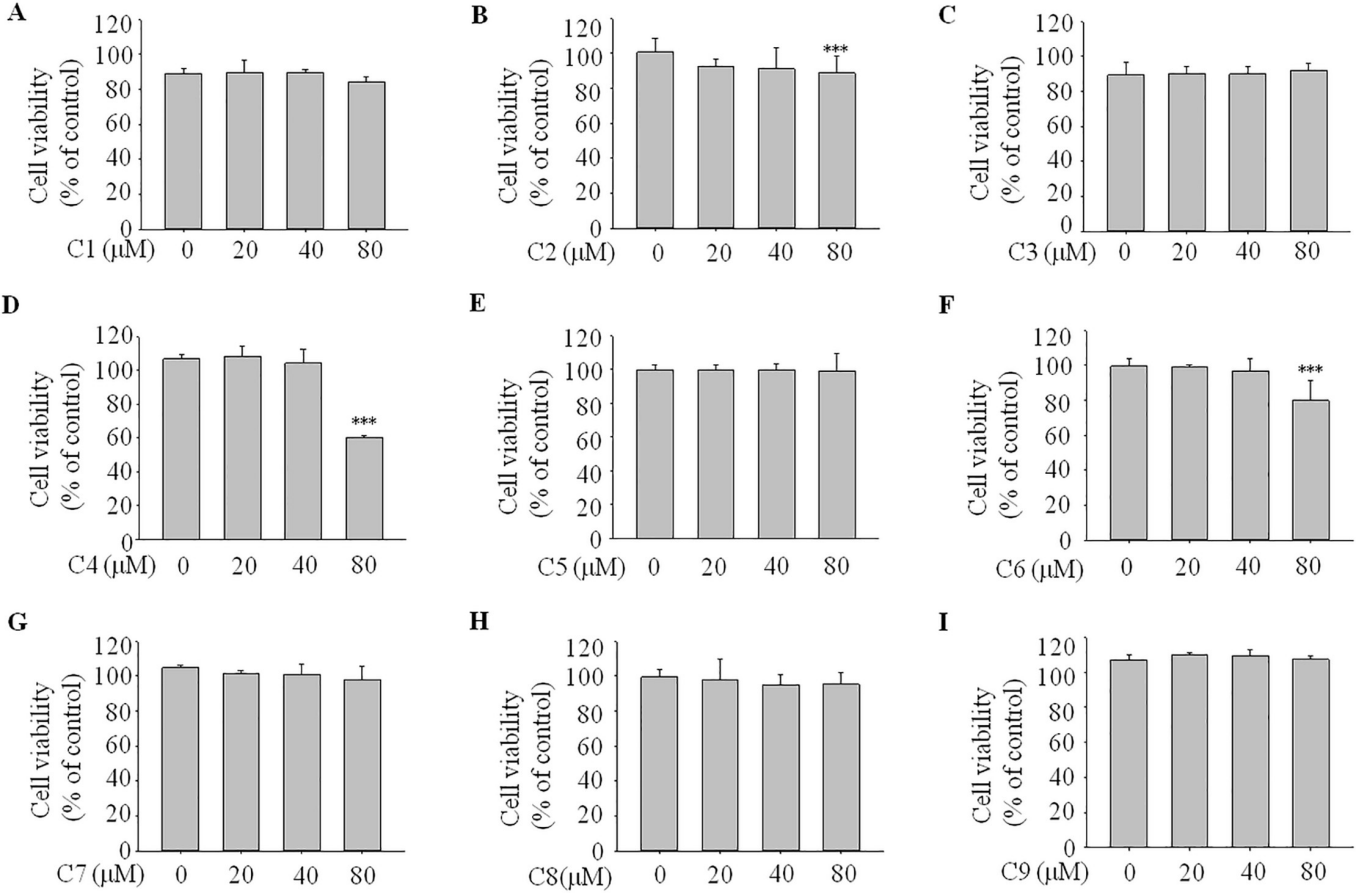
Effects of the nine compounds on viability of 3T3-L1 cells. We treated 3T3-L1 cells with 30 compounds for 96 h at concentrations of 0, 10, 20, 40, and 80 μM. The cytotoxicities of these compounds were determined by MTT assay. Parts A–I correspond to the cell viabilities of compounds **1**–**9**. Data represent the means ± standard deviations of three separate experiments. *** *P* <0.001 compared with the 0-μM group.

To examine cellular toxicity, we treated 3T3-L1 cells with each of the nine compounds for 96 h at various concentrations (0−80 μM). An MTT assay indicated that the concentrations of 0–80 μM of compounds **1**, **3**, **5**, **7**, **8**, and **9** and concentrations of 0–40 μM of compounds **2**, **4**, and **6** showed no toxicity (Fig 3). We employed 40 μM of compounds **1**, **3**, **5**, **7**, **8**, and **9** and 20 μM of compounds **2**, **4**, and **6** in the subsequent experiments.

### Inhibitory effects on lipid accumulation from Oil-Red O staining in 3T3-L1 cells

We divided 3T3-L1cells as follows: a normal control group (CON); a differentiated control (negative control) group treated with differentiation medium (DM); a differentiated control group treated with differentiation medium plus pioglitazone (PIO); and nine individual treatment groups treated with the nine compounds alone. We used the DM group to confirm the success of cell differentiation. Pioglitazone can significantly promote the differentiation of 3T3-L1 preadipocytes [28], and we employed that to demonstrate that there was no significant difference between the PIO and DM groups.

**Fig 4:**
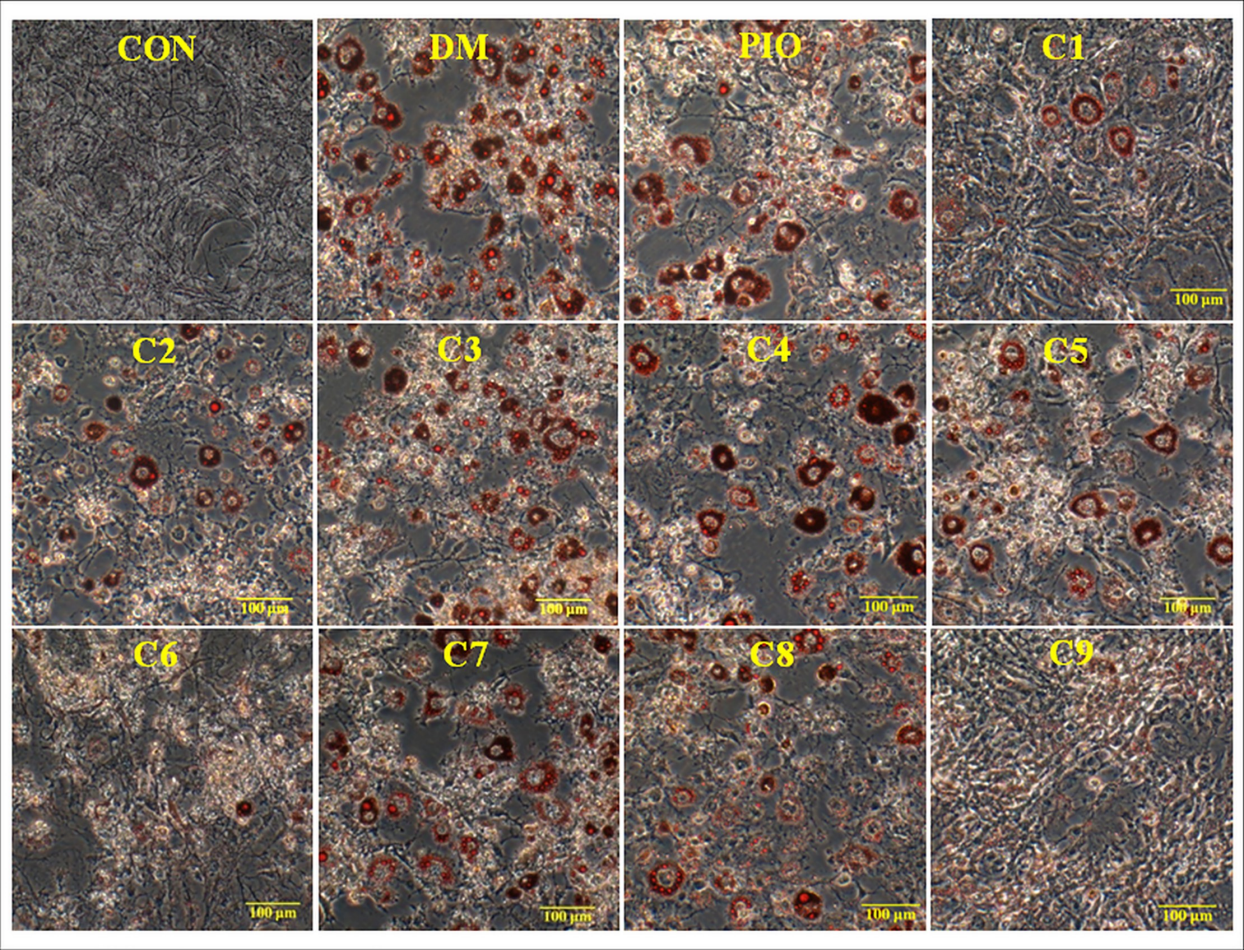
Inhibitory effects of the nine compounds on lipid accumulation from oil red O staining in 3T3-L1 cells. We evaluated lipid accumulation using oil red O staining in 3T3-L1 cells. We divided the cells as follows: a normal control group (CON); a differentiated control group treated with differentiation medium (DM); a differentiated control group treated with differentiation medium plus pioglitazone (PIO); and nine individual treatment groups treated with the nine compounds alone.

The results of oil red O staining showed that compared with the DM group (negative control), there was decreased accumulation of oil droplets in 3T3-L1 cells treated with compounds **1**, **6**, and **9**. The differentiation of 3T3-L1 cells was inhibited to some extent (Fig 4).

### Effects on lipid accumulation in 3T3-L1 cells

**Fig 5:**
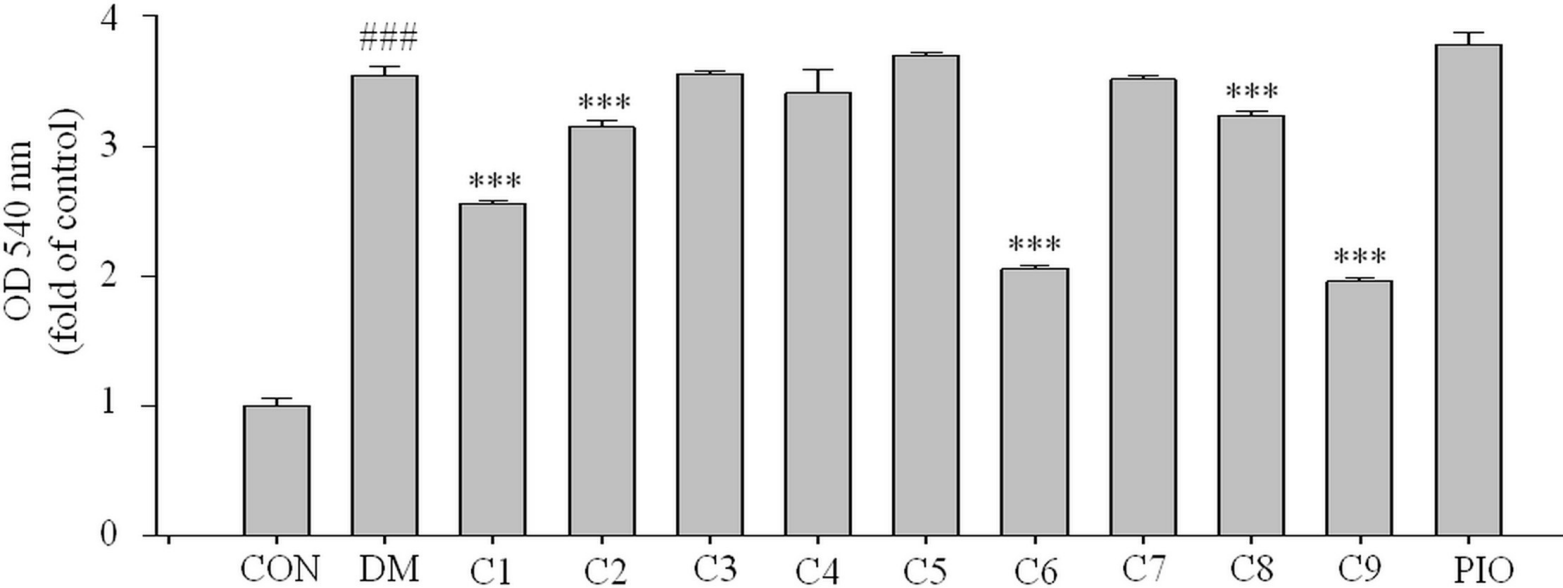
Effects of the nine compounds on lipid accumulation in 3T3-L1 cells. The 3T3-L1 cells were treated with the nine compounds respectively. After oil red O staining, we treated the cells with isopropanol and measured the lipid accumulation. Data represent the means ± standard deviations of the three separate experiments. ^###^ *P* <0.001 compared with the CON group; *** *P* <0.001 compared with the DM group. CON = normal control group, DM = differentiated control group treated with differentiation medium, PIO = differentiated control group treated with differentiation medium plus pioglitazone.

After oil red O staining, we treated the plates with isopropanol and measured the lipid accumulation levels. The absorbance values reflected differentiation of the 3T3-L1 cells. As shown in Fig 5, the absorbance values of the 3T3-L1 cells treated with compounds **1**, **6**, and **9** showed decreases to 76%, 61%, and 58%, respectively, compared with the DM group (*** *P* <0.001, respectively).

### Effects on TGs in 3T3-L1 cells

**Fig 6:**
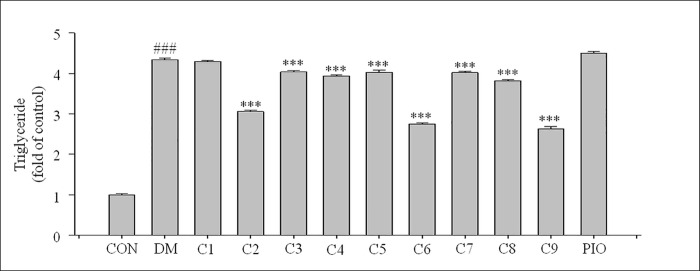
Effects of the nine compounds on triglycerides in 3T3-L1 cells. We measured triglyceride levels using the TG Assay Kit. The data represent the means ± standard deviations of three separate experiments. ^###^ *P* <0.001 compared with the CON group; * *P* <0.05, *** *P* <0.001 compared with the DM group. CON = normal control group, DM = differentiated control group treated with differentiation medium, PIO = differentiated control group treated with differentiation medium plus pioglitazone.

TGs are the most abundant lipid component in the human body, and they are used as an index of lipid-related diseases. We determined the amount of TGs in 3T3-L1 cells. We found that TG levels in cells treated with compounds **6** and **9** showed decreases to 63%, and 60%, respectively, compared with the DM group (*** *P* <0.001, respectively) (Fig 6).

All the above results indicate that compared with the DM group, compounds **6** and **9** showed the best inhibitory activities on differentiation of 3T3-L1 cells and lipid accumulation. Accordingly, we investigated their mechanisms of action by using molecular docking methods.

### Molecular docking results

**Fig 7:**
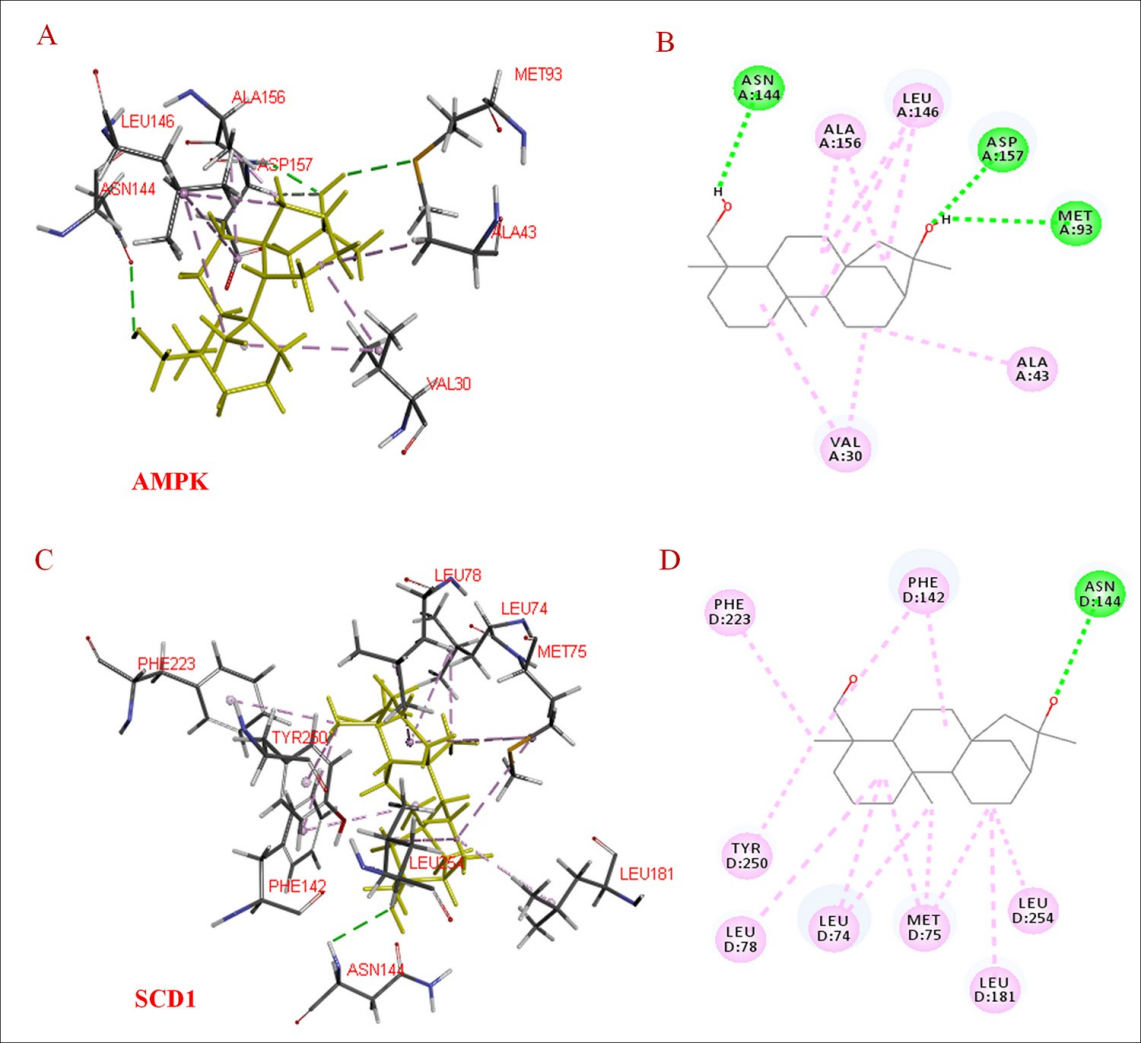
Docking interactions of compound 6 with AMPK and SCD1. A, Interactions of compound **6** with AMPK; B, 2-D view of interactions between compound 6 with AMPK; C, interactions of compound **6** with SCD1; D, 2-D view of interactions between compound **6** and SCD1. AMPK = AMP-activated protein kinase, SCD1 = stearoyl-CoA desaturase-1.

We used molecular docking methods to elucidate whether there was any interaction between compound **6** (or **9**) and AMPK (or SCD1). Compound **6** formed conventional hydrogen bonds with residues Asn-144, Asp-157, and Met-93; it formed alkyl bonds with residues Ala-156, Ala-43, Leu-146, and Val-30 when docked with AMPK (Fig 7A and 7B). Further, compound 6 formed conventional hydrogen bonds with residues Asn-144; it formed pi-alkyl or alkyl bonds with residues Phe-233, Phe-142, Try-250, Leu-78, Leu-74, Met-75, Leu-181, and Leu-254 when docked with SCD1 (Fig 7C and 7D).

**Fig 8:**
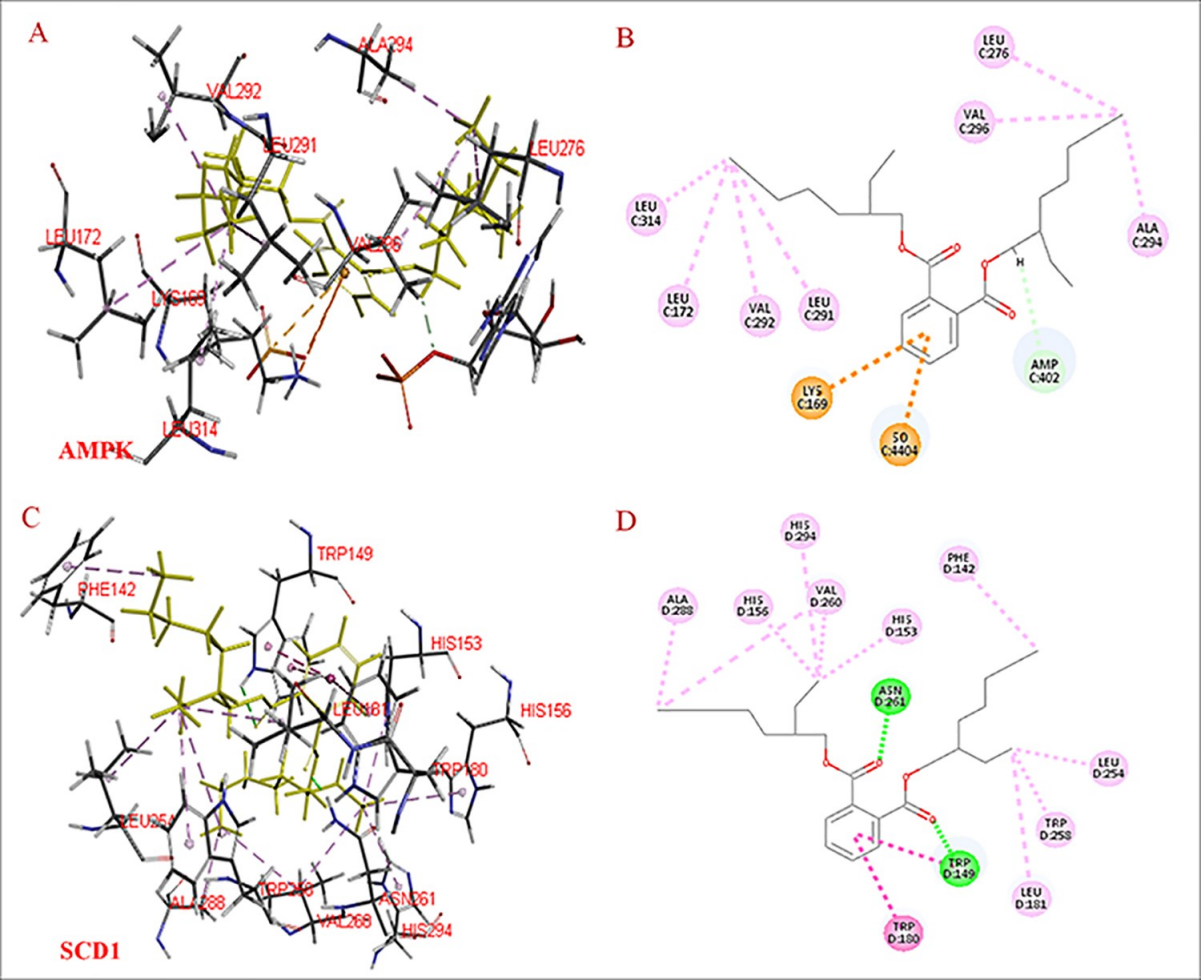
Docking interactions of compound 9 with AMPK and SCD1. A, Interactions of compound **9** with AMPK; B, 2-D view of interactions between compound **9** and AMPK; C interactions of compound **9** with SCD1; D, 2-D view of interactions between compound **9** and SCD1. AMPK = AMP-activated protein kinase, SCD1 = stearoyl-CoA desaturase-1.

We found that compound **9** formed carbon hydrogen bonds with residues Amp-402; it formed alkyl bonds with residues Leu-276, Val-296, Ala-294, Leu-314, Leu-172, Val-292, and Leu-291; and it formed pi-cation or pi-anion bonds with residues Lys-169 and So-4404 when docked with AMPK (Fig 8A and 8B). At the same time, compound **9** formed conventional hydrogen bonds with residues Asn-261 and Trp-149; it formed pi-alkyl or alkyl bonds with residues Ala-288, His-156, His-294, His-153, Val-260, Phe-142, Leu-254, Trp-258, and Leu-181; and it formed pi-pi stacked or pi-pi t-shaped bonds with residues Trp-180 when docked with SCD1 (Fig 8C and 8D).

## Discussion

To promote the development of theory and practice with TCM, it is necessary to combine traditional and innovative techniques; modern theory and technology should be applied in research efforts. TCM includes the traditional practices of all nationalities in China [3]. *Ganyancao* is a kind of Chaoyao medicine, which is a branch of TCM. Artemisinin was derived from *Artemisia annua* L. In a similar fashion, it is important for new drug research and development to identify active compounds among traditional medicines.

In previous experiments, we found that a water extract of *ganyancao* had the effect of inhibiting fat accumulation. In the present study, we extracted roots of *ganyancao* (10 kg) using pure water rather than an organic solvent. We subjected the resulting extract (1578 g) to D101 macroporous resin column chromatography with water and successively with 25%, 50%, 75%, and 95% EtOH. We isolated nine compounds from the 50%, 75%, and 95% EtOH fractions using various methods, such as macroporous resin column chromatography, reverse column (ODS-A) chromatography, and Sephadex LH-20 column chromatography, to separate the water extract. We identified the chemical structures and conducted comprehensive analyses of HR-ESI-MS and 1D, 2D NMR (such as ^1^H NMR, ^13^C NMR,HMQC, HMBC, and HR-ESI-MS): all these items were isolated for the first time in this medicinal plant.

Compounds **1**–**3** were tropone derivatives with an unusual seven-membered ring structure. Compound **1** (ganyearmcaoone A) was a new compound. Compound **3** has previously been tested for anti-tumor and anti-bacterial activities [29, 30]. However, this study was the first attempt to examine the inhibitory activities of compounds **1**–**3** on fat accumulation. Compound **1** showed potential activity with lipid accumulation in the oil red O staining experiments. Further study is required for compound **1** regarding the inhibitory effects on fat accumulation after structural modifications.

The 3T3-L1 cell line is one of the most commonly used lines for studying adipocyte differentiation in vitro [31]. Adipogenesis is accompanied by the accumulation of intracellular lipid droplets, which can be easily detected using oil red O staining [32]. The TG content is an important index of lipid accumulation. In the present study, we evaluated the inhibitory effects of the nine compounds on lipid accumulation in 3T3-L1 cells: we used photographic and quantitative assessments of the lipid content with oil red O staining and measuring TG levels. Compared with the control, compounds **6** and **9** significantly inhibited the differentiation of 3T3-L1 cells and lipid accumulation throughout this study. Our search results indicate that these two compounds have not previously been used to inhibit fat accumulation.

AMPK adjusts lipid metabolism and suppresses adipogenesis; it does so by inhibiting expression of C/EBPα, SREBP1c, and PPARγ, which are involved in adipogenesis and lipogenesis. SCD1 is an adipocyte-specific gene that is closely related to adipocyte maturation and biosynthesis of TGs [16, 17, 33]. Molecular docking (a computer simulation methodology) is used to determine the conformation of a protein-ligand complex and examine the position of a compound at any protein-binding site [18–20]. In the present study, we used molecular docking methods to elucidate whether there was any interaction between compound **6** (or **9**) and AMPK (or SCD1). We found that compounds **6** and **9** may efficiently bind to and activate AMPK and its downstream kinase (SCD1), thereby inhibiting lipid accumulation.

## Conclusions

We isolated one new (ganyearmcaoone A, **1**) and eight known compounds (**2**–**9**) from a water extract of the dried roots of *Potentilla longifolia*; all of the compounds were isolated for the first time from this plant. We elucidated the chemical structures of these compounds using 1D, 2D NMR, and HR-ESI-MS analysis. The structures of the seven-membered ring of compounds **1**–**3** (including ganyearmcaoone A) were novel; further pharmacological study is needed in the future. We evaluated the inhibitory effects of the nine compounds on lipid accumulation in 3T3-L1 cells; we did so using photographic and quantitative assessments of the lipid content with oil red O staining and by measuring TG levels. Compared with the negative control, compounds **6** and **9** significantly inhibited differentiation of 3T3-L1 cells and lipid accumulation. Molecular docking results showed that compounds **6** and **9** may efficiently bind to AMPK and its downstream kinase (SCD1), thereby inhibiting lipid accumulation. All our results show that *ganyearmcao* and its components may play an important role in treating diseases related to lipid accumulation in the future.

## Supporting information

S1 File (DOCX)

S1 Data (XLSX)
