# Supplementary material for: Inhibitory effects of compounds from the roots of Potentilla longifolia on lipid accumulation
Source: PLoS One. 2020 Sep 9;15(9):e0238917. doi: 10.1371/journal.pone.0238917 (PMC7480838; doi:10.1371/journal.pone.0238917)
Supplement: S1 File — (DOCX) [file pone.0238917.s001.docx]

**Title:** Inhibitory effects of compounds from the roots of *Potentilla longifolia* on lipid accumulation

| **No.** | **Contents** | **Page** |
| --- | --- | --- |
| 1 | **S1 Fig.** ^1^H NMR spectrum of compound **1** in CD_3_OD (500MHz). | 2 |
| 2 | **S2 Fig.**^13^C NMR spectrum of compound **1** in CD_3_OD (125MHz). | 3 |
| 3 | **S3 Fig.** HMQC spectrum of compound **1** in CD_3_OD | 4 |
| 4 | **S4 Fig.** HMBC spectrum of compound **1** in CD_3_OD | 5 |
| 5 | **S5 Fig.** HR-ESI-MS spectrum of compound **1** | 6 |


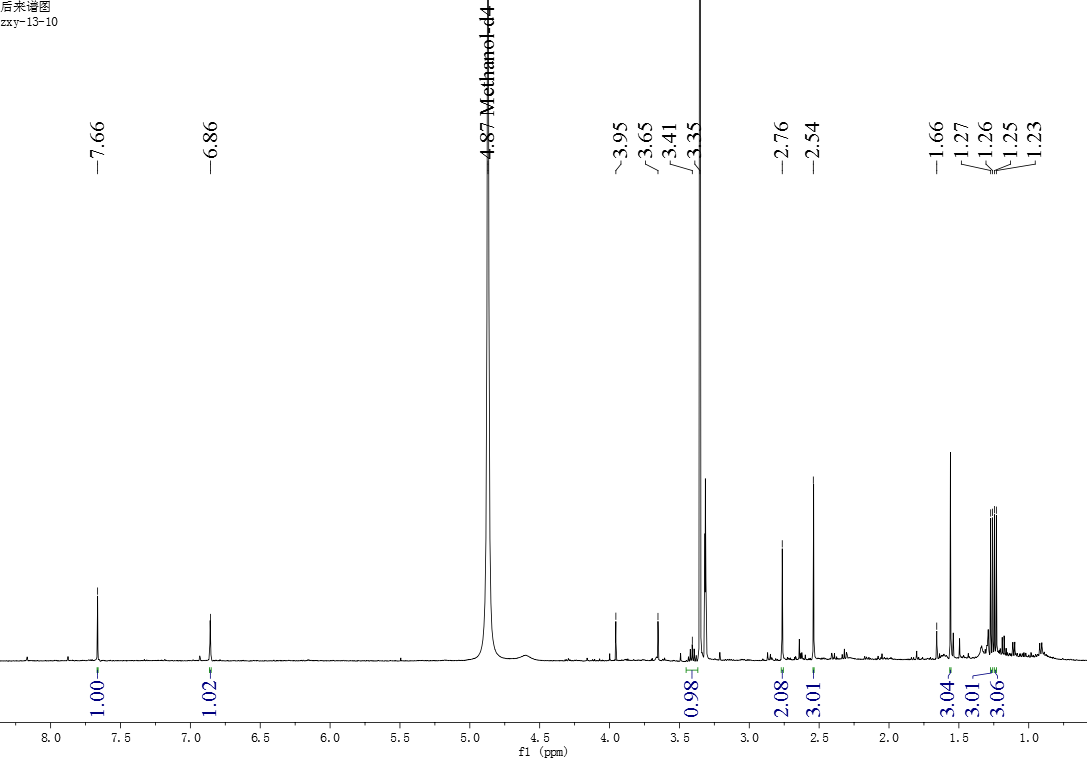


**S1 Fig.** ^1^H NMR spectrum of compound **1** in CD_3_OD (500MHz).


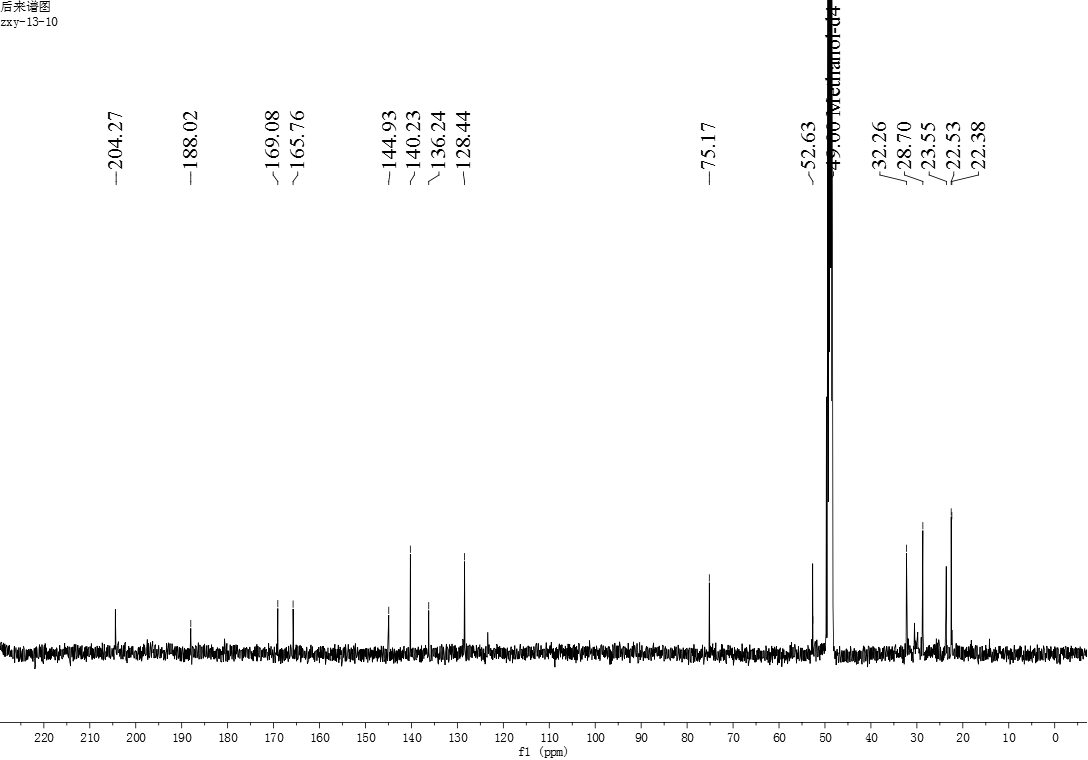


**S2 Fig.**^13^C NMR spectrum of compound **1** in CD_3_OD (125MHz).


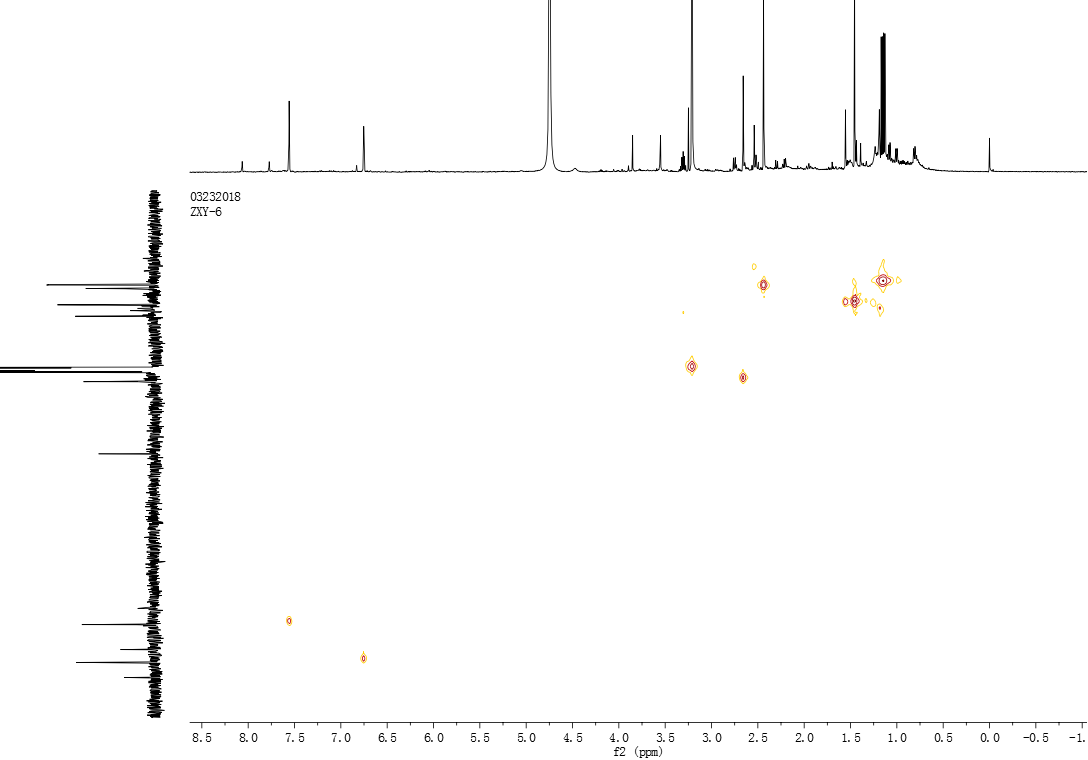


**S3 Fig.** HMQC spectrum of compound **1** in CD_3_OD.


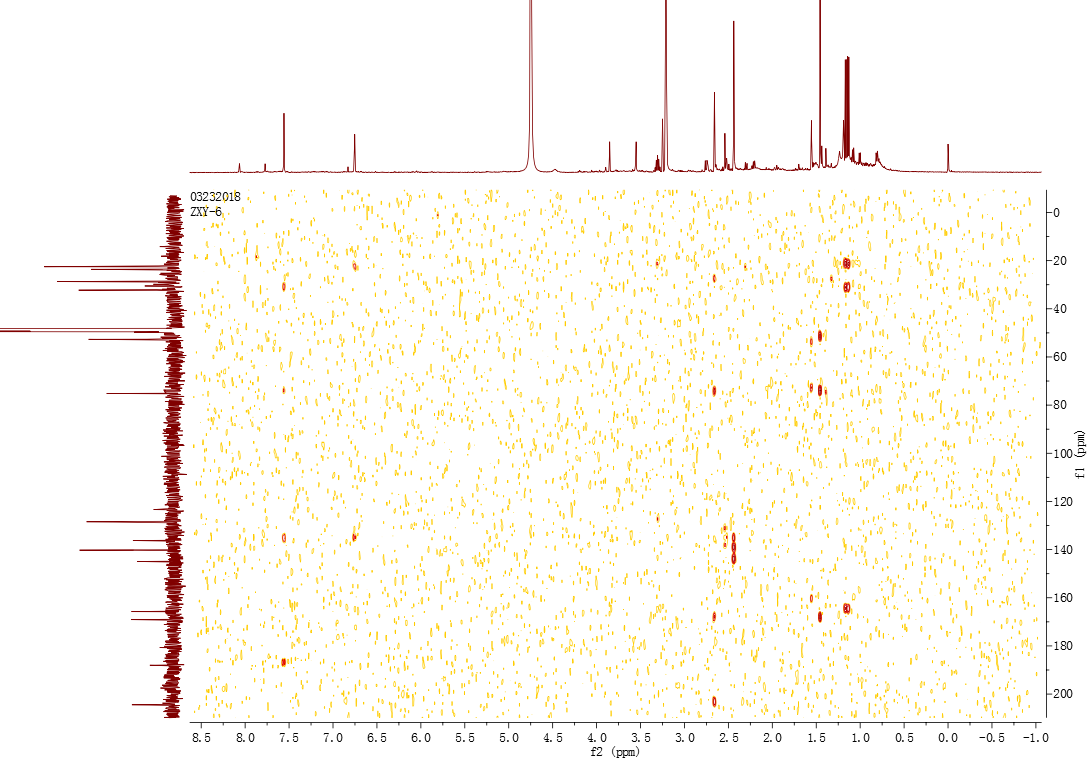


**S4 Fig.** HMBC spectrum of compound **1** in CD_3_OD.


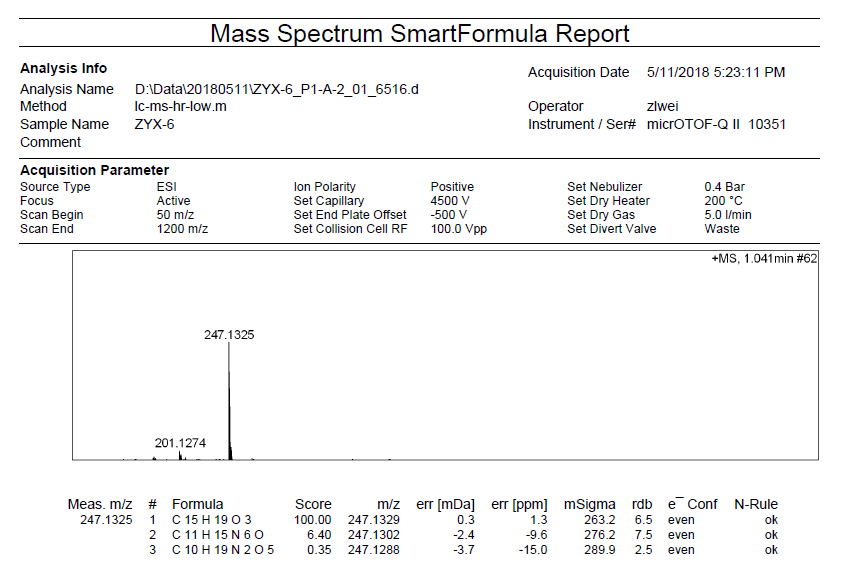


**S5 Fig.** HR-ESI-MS spectrum of compound **1**.
